# Characterization and evaluation of a novel polylactic acid-made bioreactor for large-scale adherent cell expansion

**DOI:** 10.1007/s00253-026-13880-4

**Published:** 2026-05-22

**Authors:** Björn Boshof, Alena Hüppner, Johanna Eichberg, Henning Reyer, Lukas Käßer, Anne Kölsch

**Affiliations:** 1 Green Elephant Biotech GmbH, Giessen, Germany; 2 TRON – Translational Oncology at the University Medical Center of the Johannes Gutenberg University gGmbH, Mainz, Germany

**Keywords:** Large-scale cell culture, Polylactic acid, HEK-293 cells, B16-F10 cells, CT-26 WT cells, Sustainable bioprocessing

## Abstract

**Abstract:**

Efficient large-scale expansion of adherent mammalian cells is essential for the production of recombinant proteins, viral vectors, and vaccines. Conventional multilayer flasks rely on scale-out approaches, require extensive manual handling, and lack monitoring process control compared to bioreactors. Here, we evaluate the CellScrew®, a dynamic 2D culture system available in three sizes ranging from 851 to 10,197 cm^2^, in which cell culture surfaces are continuously rotated, generating controlled hydrodynamic conditions that enhance mass transfer while reducing material consumption. Hydrodynamic conditions and oxygen transfer were characterized, with k_L_a values up to 6.69 ± 0.10 h^−1^. In this context, we assessed cell attachment, proliferation, metabolism, and harvest performance using human embryonic kidney 293 (HEK-293), murine melanoma (B16-F10), and murine colon carcinoma (CT-26 WT) cells, which serve as established model systems for biotherapeutic manufacturing applications such as viral vectors and vaccine production. The system supported cell densities up to 7.7 × 10E5 cells per cm^2^ (HEK-293), 8.7 × 10E4 cells per cm^2^ (B16-F10), and 1.1 × 10E5 cells per cm^2^ (CT-26 WT) with viabilities above 92%, comparable to multilayer flasks. Glucose consumption and lactate accumulation indicated stable metabolic activity. Mechanical harvesting enabled recovery efficiencies exceeding 89% while reducing detachment reagent usage by 67%. Overall, these results suggest that the CellScrew® can support robust adherent cell expansion with potential improvements in process efficiency and reduced resource consumption, indicating that it may represent a scalable and potentially more sustainable alternative to traditional multilayer platforms.

**Key points:**

• *CellScrew® reaches up to 0.77 ± 0.14 × 10E6 cells per cm*^*2*^* (cell-line dependent).*

• *Harvest efficiency with 67% less detachment reagent use.*

• *High oxygen transfer (kLa up to 6.69 ± 0.10 h−1) enables efficient culture.*

**Supplementary Information:**

The online version contains supplementary material available at 10.1007/s00253-026-13880-4.

## Introduction

Large-scale adherent mammalian cell expansion is crucial for viral vector manufacturing, vaccine development and manufacturing, and advanced in vitro models. However, conventional scale-up using multilayer vessels or roller bottles is labor-intensive, space-demanding, and associated with substantial single-use plastic waste (van der Loo and Wright 2016). In addition, standard static laboratory systems increase the risk of contamination due to a large number of open manipulation steps required during operation (van der Loo and Wright 2016). Multilayer stack systems like the Nunc™ Cell Factory™ or the Corning® HYPERStack® can reach 25,280 cm^2^ and 18,000 cm^2^ in a single system. However, scaling within this format can reduce lentiviral titers, with Stibbs et al*.* (Stibbs et al. 2024b) reporting a drop in titer when moving from a 1,264 cm^2^ to a 6,320 cm^2^ multilayer flask. While microcarrier-based stirred systems provide high surface-to-volume ratios and, by increasing the culture volume, surface area beyond 10 m^2^ in moderately sized vessels (e.g. Cytodex 1 at 3 g/L resulting in 13,200 cm^2^/L) they introduce challenges related to shear stress, oxygen transfer, and process control (Tsai et al. 2020). Finally, various fixed-bed bioreactors are established in bioproduction. These systems immobilize anchorage-dependent cells on a stationary matrix while the growth medium is perfused through the bed. Systems like the Cytiva iCELLis^TM^ or the Corning® Ascent® offer surface areas up to 500 m^2^ (iCELLis 500 +) and 100 m^2^ currently available for Ascent®. Fixed-bed bioreactors deliver high cell densities but require substantial capital expenditure for controllers, pumps, sparging, and utility infrastructure. Additionally, the difficulty of efficiently detaching and recovering viable cells from the matrix is known and restricts these systems from cell-based products although protocol optimization can improve harvest efficiency and viability (Goral et al. 2024; Weber et al. 2010). Enzymatic cell detachment plays a limited role during the final production phase of viral vectors, vaccines, or recombinant proteins, where the product is harvested from the supernatant or through cell lysis, but remains a critical unit operation during seed-train expansion and in workflows where the cell itself is the product, where cumulative reagent consumption scales with the number of serial passages. Beyond bioreactor architecture, construction materials are also receiving increasing attention: petroleum-derived polystyrene, polypropylene, polyethylene, and polycarbonate dominate today’s single-use equipment, while dissolvable microcarriers, plant-based materials, and new culture concepts are gaining importance under rising sustainability requirements (Handral et al. 2023; To et al. 2025). Single-use plastics contribute significantly to laboratory waste, much of which is non-recyclable due to biological contamination, accounting for approximately 2% of global plastic waste (Urbina et al. 2015). Recent initiatives driven by the European Green Deal and laboratory sustainability programs highlight the urgent need for more resource-efficient and circular solutions (Freese et al. 2024). Biobased and biodegradable polymers such as polylactic acid (PLA) have therefore emerged as promising alternatives to petroleum-derived plastics, offering reduced carbon footprints and the potential to mitigate plastic waste without compromising sterility or optical performance (O Loughlin et al. 2024). Integrating such materials into adherent culture platforms is conceptually motivated by these sustainability considerations; a quantitative life cycle or environmental impact assessment of the CellScrew® was, however, beyond the scope of the present study.

The CellScrew® integrates bio-based material choice (PLA), additive manufacturing of the internal geometry, and a rotating 2D culture design within a single roller-bottle-sized vessel. The internal structure consists of concentric cylindrical growth surfaces arranged around a central tube. Integrated Archimedes screws transport medium between the cylinders as the vessel rotates horizontally on a roller device, resulting in homogeneous medium distribution and consistent nutrient supply under low shear forces. The central tube enables constant liquid flow and allows medium handling with standard serological pipettes. This design combines mixing, oxygen transfer, and a high growth surface area within a single vessel. It therefore eliminates the need for impellers, spargers, or an external conditioning loop, as required in stirred-tank or fixed-bed systems. It comes in three different sizes ranging from 851 cm^2^ up to 10,197 cm^2^. These surface areas are in range of the size of standard roller bottles that start at ~ 850 cm^2^ up to comparable single units of multilayer stacks, for example the Nunc™ Cell Factory™ 10 with 6,320 cm^2^, or ~ 500 mL culture medium with 3 g/L Cytodex I microcarrier (resulting in ~ 9,000 cm^2^). When using the CellScrew® instead of smaller scale standard systems, the reduction of the number of vessels brings benefits, such as reduced manual labor, clean room space, a reduced risk of contamination, and less frequent detachment procedures. The design further enables reduced use of enzymatic detachment agents, potentially improving cell vitality and lowering reagent consumption (Yang et al. 2010).

Transitioning from static to dynamic adherent systems requires careful balancing of oxygen transfer and shear stress. The volumetric mass transfer coefficient (k_L_a) is a key parameter for evaluating oxygen supply (Tisu and Pavko 2010). Although this framework is well established for suspension cultures, translating it into adherent systems introduces additional complexity: oxygen must be supplied efficiently while avoiding excessive shear forces that could impair cell attachment or viability (Cherry and Papoutsakis 1988). This balance between gentle mixing and adequate gas transfer is a central characteristic of the CellScrew® system, making it a resource-efficient yet biologically compatible alternative to conventional multilayer flask-based workflows. Established dynamic systems such as roller bottles operate at low shear but remain limited in scalability and process intensification, since shear depends on vessel diameter, rotational speed, and medium volume (Bellani et al. 2020).

Human embryonic kidney (HEK-293) cells are widely used for viral vector production due to their human-like post-translational modifications and high transfectability, yet large-scale adherent cultivation remains resource-intensive (Tan et al. 2021; Rout-Pitt et al. 2018; Goh and Ng 2018). Although suspension-adapted variants exist, adherent cultivation remains standard for many clinically relevant vector systems. Additionally, murine cancer cell lines such as the colon carcinoma line CT-26 Wild-Type (CT-26 WT) and the melanoma line B16-F10 are extensively used in preclinical oncology research, including chemotherapy, immunotherapy, and drug development (Castle et al. 2012; Kreiter et al. 2015; Sahin et al. 2017). Their frequent use in *in-vivo* tumor models further underscores the demand for scalable adherent culture systems.

This study aims to characterize and evaluate the CellScrew® as a scalable and resource-efficient platform for adherent mammalian cell culture. To our knowledge, this represents the first comprehensive and systematic evaluation of this platform under biologically relevant conditions, encompassing engineering characterization and multi-cell-line cultivation. We hypothesize that the rotation-driven, PLA-based design provides adequate oxygen transfer and homogeneous medium distribution at low shear rates, supporting robust attachment and growth of adherent cells across multiple cell lines, while enabling efficient cell harvest with reduced reagent consumption. Therefore, we first characterize key bioprocess parameters, including mixing time, oxygen transfer and shear stress. We further assess the attachment, proliferation, and metabolic activity of HEK-293 cells, followed by an evaluation of murine cell lines (CT-26 WT, B16-F10) to determine the broader applicability of the system across different adherent cell lines. Finally, we quantify the potential reduction in detachment reagents enabled by the CellScrew® design, addressing both process efficiency and sustainability. Together, these results provide a comprehensive overview of the technical capabilities and practical advantages of the CellScrew® for modern adherent bioprocessing.

## Materials and methods

### Determination of mixing time in the CellScrew® system

An aqueous dye solution was prepared by diluting commercially available food coloring (ASIN B08NPRQTFD, emerald green, Wenburg, EU) in distilled water. The CellScrew® was filled with 83 mL (~ 0.098 mL/cm^2^, CellScrew® mini), 600 mL (~ 0.098 mL/cm^2^, CellScrew® 6 K) and 1000 mL (~ 0.098 mL/cm^2^, CellScrew® 10 K) of water before each experiment. The CellScrew® was rotated clockwise at 0.5, 1, and 2 rpm on a roller device (88881004, ThermoFisher Scientific, Darmstadt, Germany) at an inclination angle of 10°. For mixing time determination, 2 mL (CellScrew® mini), 5 mL (CellScrew® 6 K) and 10 mL (CellScrew® 10 K) of the prepared food coloring solution was added to the rotating system. Mixing time was defined as the time required to achieve a visually homogenous distribution of the dye throughout the entire liquid volume. Dye volumes were chosen pragmatically to ensure reliable visual detection in each vessel size rather than to maintain a constant dye-to-volume ratio, but the same visual endpoint was applied in all cases. All measurements were performed in triplicate (*n* = 3) for each rotation speed and vessel size.

### Determination of the volumetric oxygen transfer coefficient (k_*L*_a)

The volumetric oxygen transfer coefficient (k_L_a) was determined using the gassing-out method. Briefly, Aqua destillata (A.dest.) at room temperature (21–23 °C) was stripped of dissolved oxygen by sparging with nitrogen at 1 bar until a stable plateau below 3% air saturation was reached. The headspace of the CellScrew® was then flushed with air (21% oxygen) at 1 bar for 10 s, and the subsequent increase in dissolved oxygen concentration was recorded using an optical dissolved oxygen sensor (VisiFerm RS485-ECS, Hamilton, Bonaduz, Switzerland) via Hamilton ArcAir software (version 3.9.2) at a 6 s sampling interval. Nitrogen and air were supplied without a mass flow controller. The maximum dissolved oxygen concentration (c*) was adjusted to the measured temperature of each experiment based on the oxygen solubility data of Benson and Krause (Benson and Krause 1984). The k_L_a value was calculated from the slope of the logarithmic reoxygenation curve according to the standard mass transfer equation. Each condition was measured in triplicate (n = 3) per rotation speed and vessel size.

### Calculation of shear rates in the CellScrew®

Shearing and shear rates are further key parameters for bioreactor characterization. The CellScrew® vessel’s novel geometry differs from stirred tank bioreactors, making a theoretical approach to shear rates necessary. Miller et al*.* introduced a prediction of non-Newtonian flow behavior in ducts of unusual cross section (Miller 1972), which is applicable to the internal structure of the CellScrew®. The Archimedes screws and the cylinders form rectangular ducts which are converted in the said publication. The shape factor λ can be calculated by using the width (*a* = 0.01 m) and height (*b* = 0.005 m) of the wall structures inside the CellScrew®. These values are identical for all CellScrew® sizes and lead to (Chemical Engineers’ Handbook 1963):

1 $$\lambda =\frac{24.0}{[{\left(\frac{1.0.351b}{a}\right)\left(1+\frac{b}{a}\right)]}^{2}}$$

λ is the dimensionless shape factor. Parameter *a* represents the width in meters (m), while *b* represents the height, also given in meters (m). This leads to λ = 15.7 for all three sizes of the CellScrew®. Subsequently the average wall shear stress can be calculated:

2 $$\overline{\tau }=\eta \left[\frac{Q\lambda }{2A{D}_{h}}\right]$$

$\overline{\tau }$ is the average wall shear stress in newtons (N). $\eta$ is the viscosity in pascal-seconds (Pa*s). Q is the volumetric flow rate in cubic meters per second (m^3^*s⁻^1^). $\lambda$ is the shape factor. A is the cross-sectional area in square meters (m^2^), and D_h_ is the hydraulic diameter in meters (m). Finally, the average wall shear rate can be calculated using:

3 $$\overline{\gamma }=\frac{Q\lambda }{2A{D}_{h}}$$

$\overline{\gamma }$ is the average wall shear rate in seconds⁻^1^ (s⁻^1^). η is the viscosity in pascal-seconds (Pa*s). Q is the volumetric flow rate in cubic meters per second (m^3^*s⁻^1^). λ is the shape factor. A is the cross-sectional area in square meters (m^2^), and D_h_ is the hydraulic diameter in meters (m).

Due to the internal structure of the CellScrew®, the highest shear rate will always occur in the most outer spiral for the respective size (spiral 6 for CellScrew® 6 K and 10 K, spiral 2 for CellScrew® mini). In this approach, the actual shape factor λ and the worst-case scenario (highest shear rate) of b/a = 0 were calculated.

### Cell culture

Human Embryonic Kidney 293 (HEK-293) cells were obtained from DSMZ (ACC 305, RRID: CVCL_0045, Braunschweig, Germany) and cultivated in RPMI 1640 (P04-16500) supplemented with 25 mM HEPES (P05-01100) and 10% (v/v) fetal bovine serum (FBS, P40-37500) at 37 °C in a humidified 5% CO_2_ atmosphere. Cells were passaged twice per week using Trypsin/EDTA (P10-024100) with a seeding density of 6.6 × 10E4 cells/cm^2^. HEK-293 cells were washed once with ~ 0.06 mL/cm^2^ Dulbecco's phosphate-buffered saline (DPBS, w/o Ca and Mg, P04-36500) before trypsinization with ~ 0.03 mL/cm^2^ Trypsin/EDTA for 5 min. The enzymatic reaction was stopped using ~ 0.03 mL/cm^2^ serum-containing medium, and cells were centrifuged at 200 × *g* for 5 min. Cells were counted using a Neubauer improved counting chamber, and 6.6 × 10E4 cells/cm^2^ were seeded into the desired format. All reagents were purchased from PAN-Biotech GmbH, Aidenbach, Germany.

The murine melanoma cell line B16-F10 (CRL-6475, RRID: CVCL_0159, C57BL/6 background, ATCC, Manassas, VA, USA) and the murine colon carcinoma cell line CT-26 Wild-Type (CT-26 WT; CRL-2638, RRID: CVCL_7256, BALB/c background, ATCC, Manassas, VA, USA) were used in this study. B16-F10 cells were maintained in DMEM (AL007S, HiMedia Laboratories GmbH, Modautal, Germany) supplemented with 10% (v/v) FBS Superior stabil (FBS.S 0615, Bio&Sell, Nuremberg, Germany), while CT-26 WT cells were kept in HiGlutaXL RPMI medium 1640 (AL028G, HiMedia Laboratories GmbH) supplemented with 10% (v/v) FBS superior stabil. Both cell lines were cultured in a humidified environment with 5% CO₂ at 37 °C and subcultured every 2–3 days. For routine passaging, B16-F10 cells were seeded at 1.5 × 10E4 cells/cm^2^ (2-day passage) or 0.75 × 10E4 cells/cm^2^ (3-day passage), and CT-26 WT cells were seeded at 2 × 10E4 cells/cm^2^ (2-day passage) or 1 × 10E4 cells/cm^2^ (3-day passage). Both murine cell lines were washed once with ~ 0.1 mL/cm^2^ Dulbecco's phosphate-buffered saline (DPBS, w/o Ca and Mg,TL1006, HiMedia Laboratories GmbH) before cell detachment with ~ 0.05 mL/cm^2^ Accutase® 1X Accutase® enzymes (TCL075, HiMedia Laboratories GmbH) for 5 min. Cultures were routinely monitored microscopically for microbial contamination. Mycoplasma contamination of all cell lines was excluded by PCR-based testing (Eurofins, Planegg, Munich, Germany), and cell line identity for murine cell lines was confirmed by STR profiling (Microsynth, Göttingen, Germany).

### Attachment kinetics of cells in different cell culture vessel formats

To compare the attachment behavior of HEK-293 in the CellScrew® and a roller bottle (Cellmaster, 850 cm^2^ Greiner Bio One GmbH, Frickenhausen, Germany), cells were seeded at a density of 0.8 × 10E5 cells/cm^2^. Roller bottle and CellScrew®mini were incubated in the Incudrive D-I (Schuett-Biotec, Göttingen, Germany) at 37 °C in a 5% CO_2_ atmosphere at 0.5 rpm. For B16-F10 and CT-26 WT cells, inoculums of 5 × 10E3 cells/cm^2^ and 1 × 10E4 cells/cm^2^ were used, respectively. At 1, 2, 3, 4, 5, 6, and 24 h post-seeding, samples of 1 mL were taken carefully, without changing the angle or interrupting the rotation of the vessels to avoid detachment of loosely attached cells. For HEK-293, an additional sample was taken at 26.5 h. Where necessary, samples were concentrated by centrifugation (400 × g, 5 min or 300 × g, 4 min depending on the cell line) and resuspension in a smaller volume prior to cell counting using a Neubauer improved counting chamber. Cell attachment was quantified by normalizing the number of adherent cells to the initial seeding density, which was defined as 0% attached cells. Subsequent time points were expressed as the percentage of cells attached relative to this baseline. All attachment experiments were performed in triplicate (*n* = 3).

### Growth curve of cells in the CellScrew® mini

All cell lines were plated in CellScrew® mini at a defined cell concentration (see seeding densities above). After 24, 48, 72, and 96 h, the B16-F10 and CT-26 WT were harvested using Accutase (BS.L2193, Bio&Sell) while HEK-293 were harvested using Trypsin/EDTA. Cell counts and viability determination were performed using a Neubauer counting chamber. The supernatant was taken to check for glucose and lactate levels. In addition, cells were monitored for bacterial or fungal contamination at each harvest time point. With the CellScrew® mini, it was not possible to perform a direct visual microscopic examination. Therefore, cell culture supernatant was transferred to deep-well plates to check for any abnormalities. The evaluation was carried out in technical triplicate.

### Lactate and glucose measurement

An enzymatic amperometric method was used to measure lactate and glucose concentrations in cell culture supernatants with the Biosen C-line (EKF Diagnostics, Barleben, Germany). The device uses chip sensors based on enzymatic reactions (glucose oxidase for glucose and lactate oxidase for lactate), combined with electrochemical detection (amperometry). The cell culture supernatant was mixed with hemolysis solution (EKF Diagnostics, Barleben, Germany) in a pre-filled reaction vessel. The sample was vortexed before the measurement was conducted.

### Reduction or replacement of the detachment reagent

Both murine cell lines were plated on 12-well plates (12-well clear bottom TC-treated, Corning, Kaiserslautern, Germany) at a defined cell concentration (1.5 × 10E4 cells/cm^2^ for B16-F10 cells and 2 × 10E4 cells/cm^2^ for CT-26 WT cells, respectively). After 72 h, the cells were detached using 100%, 80%, 60%, 40%, and 20% Accutase. The total volume was filled up to 1 mL with DPBS (TL1006, HiMedia Laboratories GmbH). In addition, both cell lines were detached using 100% of the plant-based detachment reagent, EnVzyme™ Super (TCL153, HiMedia Laboratories GmbH). After equal detachment time, the cell numbers and viability were determined using a Neubauer counting chamber. The evaluation was done in duplicates.

### Determination of harvest efficiency in the CellScrew®

For the CellScrew® mini the first harvest was done using 50 mL (~ 0.059 mL/cm^2^) of Trypsin and tapping the CellScrew® mini a total of 5 times at regular intervals around the bottle. In the second harvest, 50 mL of Trypsin was added, and the CellScrew® mini was tapped 5 × 5 (for a total of 25) times at regular intervals around the bottle. All tapping was done using a rubber mallet. Building on these results, the CellScrew® 6 K was harvested using only the reduced condition (trypsin diluted 1:3 in DPBS) at a total volume of 300 mL (~ 0.049 mL/cm^2^) and tapping the CellScrew® 6 K 5 × 5 (total of 25) times at regular intervals around the bottle. Harvest experiments in the CellScrew® 6 K were performed at five independent time points (48, 72, 96, 144, and 168 h post-seeding). Each harvest was performed once per time point (*n* = 1) and is intended as a technical, proof-of-concept evaluation of harvest feasibility and efficiency at the larger scale rather than as a fully replicated quantitative comparison. Harvest experiments in the CellScrew® mini were performed three times per condition (*n* = 3) and provide the biologically replicated reference data set.

### Statistical analysis

All quantitative data are presented as mean ± standard deviation (SD). The number of replicates (n) and whether replicates were biological or technical is indicated in the corresponding figure legends. Mixing time data were analyzed by two-way ANOVA with rotational speed and system size as factors, including their interaction. Differences in cell attachment between the CellScrew® mini and the roller bottle reference were assessed by two-way ANOVA followed by Tukey’s multiple comparisons test. *P*-values < 0.05 were considered statistically significant and are indicated in the figures (*p < 0.05; ns, not significant). All statistical analyses and graphical representations were performed using GraphPad Prism version 10 (GraphPad Software, San Diego, CA, USA).

## Results

### Mixing time and oxygen transfer

**Fig. 1:**
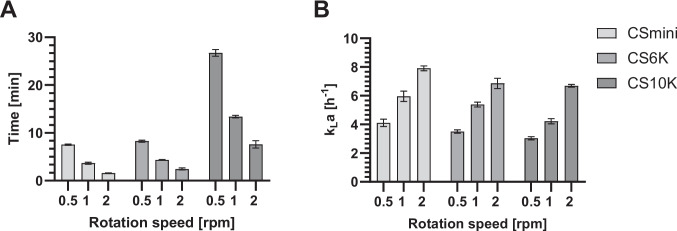
Hydrodynamic and mass transfer characteristics in bioprocessing range. (**A**) Mixing time required for homogeneous liquid distribution in CellScrew® during different rotation speeds. The minimal working volume was stained, and the time until complete visual homogenization was recorded. (**B**) Volumetric oxygen transfer coefficients (k_L_a) values were measured using gassing-out method by monitoring dissolved oxygen reoxygenation with an optical sensor and calculated from the slope of the logarithmic reoxygenation curve. Values represent the mean ± SD of *n* = 3 technical replicates. Statistical significance was determined by two-way repeated measures ANOVA. CS, CellScrew®

Mixing time and the volumetric oxygen transfer coefficient (k_L_a) are key parameters for assessing hydrodynamic performance and oxygen availability in bioprocesses. The mixing time was determined in the CellScrew® mini, CellScrew® 6 K, and the CellScrew® 10 K at 0.5, 1, and 2 rpm rotational speed (Fig. 1A). For the CellScrew® mini, mixing times decreased from 7.5 ± 0.10 min at 0.5 rpm to about 3 ± 0.17 min at 1 rpm and 1.5 ± 0.03 min at 2 rpm. In the CellScrew® 6 K, mixing times ranged from 8.3 ± 0.21 min at 0.5 rpm to 4.3 ± 0.04 min at 1 rpm to 2.3 ± 0.16 min at 2 rpm. The CellScrew® 10 K showed longer mixing times at lower rotational speeds, with 27.7 ± 0.57 min at 0.5 rpm, decreasing to 13.4 ± 0.21 min at 1 rpm and 7.5 ± 0.62 min at 2 rpm. All values are reported as mean ± SD of n = 3 technical replicates. A two-way ANOVA revealed significant effects of both rotational speed (p = 0.0003) and system size (p = 0.0002), and their interaction (p = 0.0002) on mixing time, confirming that increasing rotational speed consistently reduces mixing time across all system sizes, with the effect being more pronounced in larger systems. In contrast, mixing in static systems such as T-flasks relies exclusively on molecular diffusion. Based on Fick’s law (t ≈ L^2^/D), assuming a typical medium height of 3–5 mm and a diffusion coefficient of ~ 10^–9^ m^2^/s, the theoretical mixing time in a T-flask is estimated to be on the order of several hours. The minute-scale mixing times achieved in the CellScrew® systems are therefore negligible compared to the biologically relevant timescale of cell attachment (1–6 h), supporting the assumption of sufficient homogeneity within the system.

The oxygen transfer coefficients, or k_L_a-values, were determined in the recommended rotational speed range at 0.5, 1 and 2 rpm (Fig. 1B). For the CellScrew® 6 K a maximum k_L_a-value of 6.68 ± 0.36 h^−1^ was observed, while the maximum k_L_a-value was 6.69 ± 0.10 h^−1^ for the CellScrew® 10 K and 7.91 ± 0.17 h^−1^ for the CellScrew® mini, respectively (mean ± SD, *n* = 3 technical replicates). As expected, the k_L_a-value increases with increasing rotational speed.

### Shear rates in the CellScrew® system

In addition to mixing and oxygen transfer, shear rates were determined to further characterize the hydrodynamic conditions of the CellScrew® system (Table 1). Shear rates in CellScrew(R) systems were evaluated as a function of rotational speed for three different vessel sizes (CellScrew® mini, CellScrew® 6 K, and CellScrew® 10 K). Both panels show a clear positive correlation between rotational speed and shear rate. For all vessel sizes, increasing the rotational speed from 0.5 to 2 rpm resulted in progressively higher shear rates. Larger vessels (CS6K and CS10K) consistently exhibited higher shear rates compared to the smallest container (CellScrew® mini) at each rotational speed. In Table 1 the left column represents the actual shape factor of the CellScrew® while the right column shows the worst-case scenario of the highest possible shear rates. Notably, the shear rate increases between 1 and 2 rpm was most pronounced for the largest vessel (CellScrew® 10 K), reaching peak values of approximately 21–22 s⁻^1^ in the left panel and 14–15 s⁻^1^ in the right panel. In comparison the second half of Table 1 shows cell damaging shear rates in different culture conditions for B16-F10, HEK-293 which were used in this study as well as for MSC and iPSC which have the tendency to be shear sensitive. Except for the highest rotational speed in the CS6K and CS10K, all calculated shear rates are below these thresholds.

**Table 1:** Comparison of shear rates of the CellScrew® and other culture vessels. Shear rate values represent model-based estimates derived from a theoretical fluid dynamics model rather than direct experimental measurements

A			
RPM	Size	Shear rate in most outer spiral [1/s]	
		**0 ≥ b/a ≤ 1**	**b/a = 0**
0.5	CSmini	2	3
	CS6K	4	6
	CS10K	4	6
1	CSmini	3	5
	CS6K	7	11
	CS10K	7	11
2	CSmini	7	11
	CS6K	15	22
	CS10K	15	22
B			
Culture conditions	Cells	Shear rate [1/s]	Publication
Perfusion Suspension with TFF (tangential flow filtration)/ATF (alternating tangential flow filtration)	HEK-293	1865—9146	(Zhan et al. 2020)
Microfluidic gel	B16-F10	40	(Pandya et al. 2017)
Microcarrier spinner	MSC (mesenchymal stromal cells)	14	(Zhang et al. 2023)
Dynamic Spheroids	MSC	250—1000	(Fuentes et al. 2022)
Adherent culture with induced shear stress	iPSC (induced pluripotent stem cells)	150—1500	(Limraksasin et al. 2022)

Calculations assume non-Newtonian flow in rectangular channels with maximum shear rate at the outermost spiral. Width (a = 0.01 m) and height (b = 0.005 m) of the CellScrew® flow channel used to calculate the shear rates. CS, CellScrew®

### Attachment and growth of HEK-293 in CellScrew® mini

**Fig. 2:**
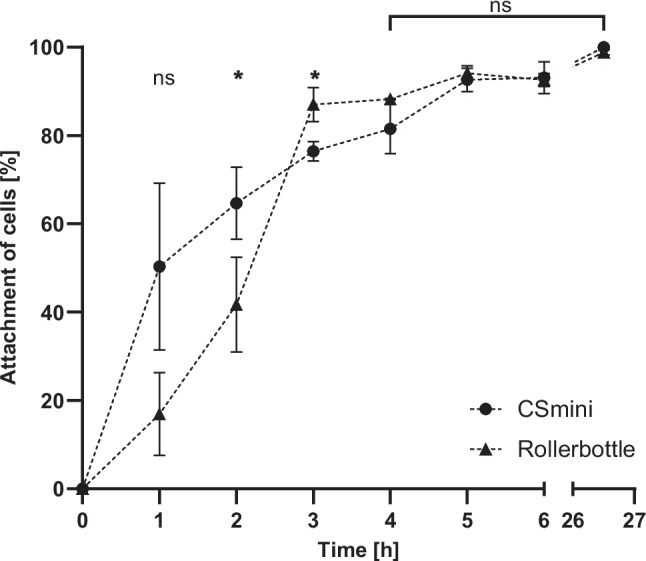
Attachment of HEK-293 cells in dynamic culture vessels. HEK-293 cells were seeded at a density of 0.8 × 10E5 cells/cm^2^, and supernatant was collected at indicated time points. Cell attachment was quantified by normalizing the number of adherent cells to initial seeding density, defined as 0% attached cells. Data are presented as mean ± SD (*n* = 3). Significant differences to the roller bottle were assessed using Turkey’s multiple comparisons test. *p < 0.05 for CSmini. Ns, not significant. CSmini, CellScrew® mini

As the CellScrew® represents a completely novel cultivation system, several parameters had to be evaluated to ensure optimal cell health and cell growth. While the growth of adherent cells is determined by various factors, attachment after inoculation is the first critical determinant of subsequent expansion. One of the advantages of the CellScrew® system is the scalability. In this study, the entry-level size CellScrew® mini was used to determine first attachment and growth behaviors. Here, HEK-293 cells were seeded in CellScrew® minis and conventional roller bottles, respectively (Fig. 2). The roller bottle was chosen as a reference system due to its mechanistic similarity to the CellScrew®: both are rotating, dynamic cultivation systems that rely on convective mixing rather than diffusion, distinguishing them from static flasks and making them directly comparable in terms of hydrodynamic conditions and mixing behavior. Cell attachment was determined by counting cells in the supernatant at the indicated time points. At 1 h, no statistically significant difference was observed between the two conditions (ns). At 2 h and 3 h, cell attachment was significantly higher on the CellScrew® compared to the roller bottle (*p* < 0.05). From 4 h onwards, attachment levels further increased for both systems and reached similarly high values. No statistically significant differences were detected between the two culture systems at later time points (5–6 h), and both conditions showed > 98% attached cells after 26 h.

**Fig. 3:**
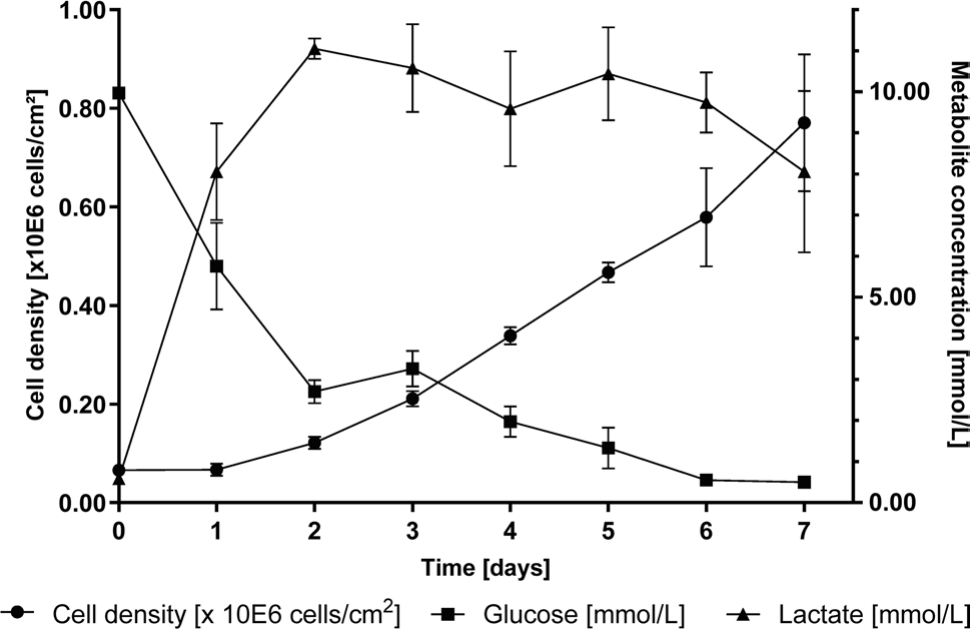
Cell growth and metabolic dynamics of HEK-293 cells in CellScrew® mini. HEK-293 cells were seeded at a cell density of 6.6 × 10E4 cells/cm^2^ in culture vessel. At indicated time points, three CellScrew® mini were harvested, supernatant collected and glucose and lactate concentration measured, respectively. Data are presented as mean (± SD) of n = 3 per time point; in some cases, error bars are smaller than the symbols and therefore not visible. CS, CellScrew®

Next, the growth of HEK-293 cells was assessed in the CellScrew® mini (Fig. 3, black circles). The cells showed robust growth and glucose consumption over time. According to the supplier information, the harvest setpoint of 2.5 × 10E5 cells/cm^2^ for the cell line was met between 72 and 96 h, with cell densities ranging from 2.1 ± 0.15 × 10E5 cells/cm^2^ to 3.4 ± 0.17 × 10E5 cells/cm^2^. At day 7, HEK-293 cells reached a maximum density of 0.77 ± 0.14 × 10E6 cells/cm^2^ demonstrating that the CellScrew® supports continued expansion well beyond the typical harvest window.

Due to its design, the CellScrew® mini cannot be examined microscopically for confluence. Instead, glucose and lactate concentrations in the cell culture supernatant were measured as indirect indicators of medium consumption and cell confluence (Fig. 3, black triangles and squares, respectively). Parallel glucose consumption and lactate accumulation during the exponential phase, followed by a reduced lactate accumulation despite continued glucose consumption, are consistent with the reported metabolic behavior of HEK-293 cells. Quantitative analysis of the lactate yield from glucose (Y_Lac/Glc_ = Δlactate/Δglucose [mol/mol]) revealed phase-specific metabolic shifts: during the initial lag phase.

(0–24 h), Y_Lac/Glc_ reached 1.77 mol/mol, reflecting a strongly glycolytic post-seeding adaption. In the subsequent exponential growth phase (24–48 h), Y_Lac/Glc_ decreased to 0.98 mol/mol, indicating a shift toward a more balanced oxidative-glycolytic metabolism. Following medium exchange, a net lactate consumption was observed (Y_Lac/Glc_ of −0.78 mol/mol from day 3 to day 7), consistent with the reported ability of HEK-293 to utilize accumulated lactate as an alternative carbon source (Liste-Calleja et al. 2015).

### Attachment of B16-F10 and CT-26 WT cell lines in CellScrew® mini

Attachment kinetics were also performed for two murine cell lines. For adhesion kinetics experiments, B16-F10 and CT-26 WT cells were seeded at a defined concentration in CellScrew® minis. At 1, 2, 3, 4, 5, 6, and 24 h post-seeding, non-adherent cells in the supernatant were quantified by aliquot sampling, while minimizing vessel movement to preserve the rotational angle. The number of adherent cells was calculated from the remaining cells in the supernatant.

**Fig. 4:**
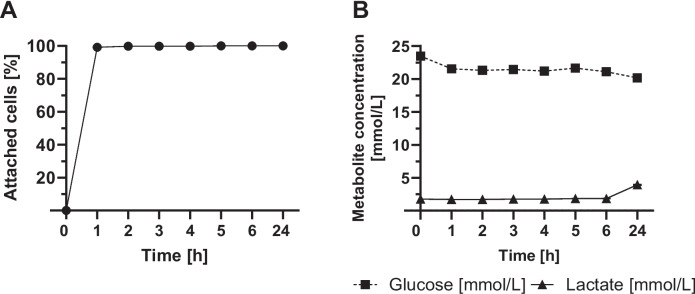
Attachment kinetics of B16-F10 cells to CellScrew® mini surface. B16-F10 cells were seeded into CellScrew® mini at a cell density of 5 × 10E3 cells/cm^2^ and attachment was monitored by taking supernatant at indicated time points. (**A**) shows attached cells over time at 2 rpm rotational speed. (**B**) shows glucose (black square) and lactate concentration (black circle) of medium over time. Values represent the mean ± SD of n = 3 biological replicates; in some cases, error bars are smaller than the symbols and therefore not visible

Figure 4 shows attachment of B16-F10 cells (A) as well as metabolite concentrations in the supernatant (B). Values represent the mean ± SD of n = 3 biological replicates. After just one hour of cultivation on the CellScrew® mini, all B16-F10 cells have adhered to the PLA surface. No cells could be detected in the supernatant. Despite the rapid adhesion of B16-F10 cells to the PLA surface, only minimal metabolic activity, as indicated by glucose and lactate concentration in the supernatant, is observed within the first one to six hours.

**Fig. 5:**
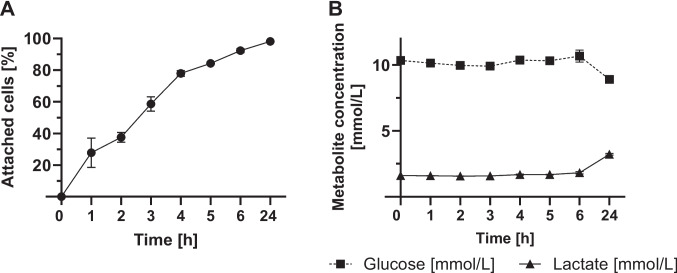
Attachment kinetics of CT-26 WT cells to CellScrew® mini surface. CT-26 WT cells were seeded into CellScrew® mini at a cell density of 1 × 10E4 cells/cm^2^ and attachment was monitored by taking supernatant at indicated time points. (**A**) shows attached cells over time at 2 rpm rotational speed. (**B**) shows glucose (black square) and lactate concentration (black triangle) of supernatant over time. Values represent the mean ± SD of n = 3 biological replicates; in some cases, error bars are smaller than the symbols and therefore not visible

Regarding the attachment of CT-26 WT cells, almost 100% of cells are attached after 24 h of cultivation, while showing minimal glucose and lactate concentration within the first six hours (Fig. 5A and B). As for B16-F10, the CT-26 WT attachment data show values represent the mean ± SD of n = 3 biological replicates.

For both cell lines, B16-F10 and CT-26 WT, a pronounced increase in metabolic activity occurs between six and 24 h. As cell density increases, glucose concentration decreases, accompanied by a proportional rise in lactate concentration. The PLA surface and the rotating system of the CellScrew® do not appear to influence the adhesion of B16-F10 and CT-26 WT cell lines.

### Growth kinetics of B16-F10 and CT-26 WT cell lines in CellScrew® mini

**Fig. 6:**
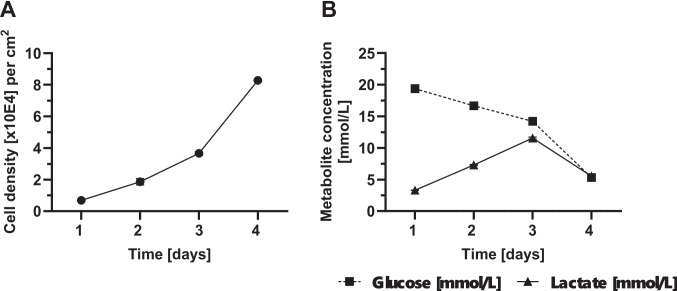
Growth kinetics of B16-F10 cells. B16-F10 cells were cultivated in CellScrew® mini at 2 rpm rotational speed for 96 h. The seeding concentration for B16-F10 cells was 5 × 10E3 cells/cm^2^. (**A**) shows the cell density [x 10E4] harvested per cm^2^ over time. (**B**) shows glucose (black square) and lactate concentration (black triangle) of supernatant over time. Data are presented as mean (± SD) of *n* = 3 per time point; in some cases, error bars are smaller than the symbols and therefore not visible

After demonstrating that both cell lines successfully adhered to the PLA surface, the next step was to assess the cell growth on the PLA surface. To this end, a growth curve was established over 96 h. Cells were harvested at 24, 48, 72, and 96 h, followed by determination of cell counts and viability. All experiments were performed in triplicates. In addition to the growth curve (Fig. 6A), lactate and glucose concentrations were monitored (Fig. 6B).

B16-F10 cells were seeded at a density of 0.5 × 10E4 cells/cm^2^ in a CellScrew® mini. As previously shown, B16-F10 cells adhered to the PLA surface within one hour. After 24 h, the cell number increased 1.4-fold, after 48 h 3.7-fold, after 72 h 7.3-fold, and after 96 h 16.5-fold, resulting in a harvest density of 8.2 ± 0.06 × 10E4 cells per cm^2^ after 96 h. During the cultivation, cell viability remains consistently above 94%. A complete medium change was performed at 72 h post-seeding. Over the cultivation period prior to the medium exchange (0–72 h), B16-F10 cells displayed a high lactate yield from glucose (Y_Lac/Glc_ = Δ lactate/Δ glucose [mol/mol]) of 1.67 mol/mol, approaching the theoretical maximum of 2 mol/mol and indicating a pronounced glycolytic phenotype.

**Fig. 7:**
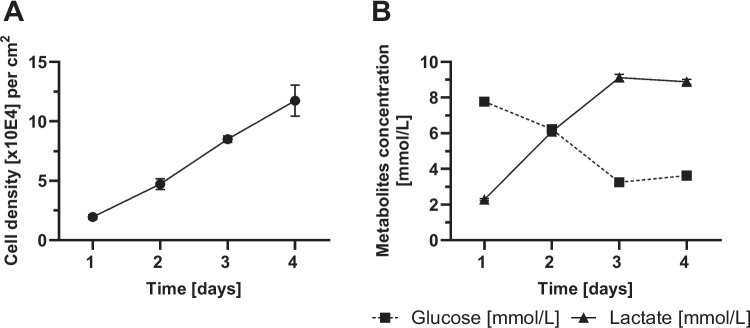
Growth kinetics of CT-26 WT cells. CT-26 WT cells were cultivated in CellScrew® mini at 2 rpm rotational speed for 96 h. The seeding concentration for CT-26 WT cells was 1 × 10E4 cells/cm^2^. (**A**) shows the cell density [x 10E4] harvested per cm^2^ over time. (**B**) shows glucose (black square) and lactate concentration (black triangle) in supernatant over time. Data are presented as mean (± SD) of *n* = 3 per time point; in some cases, error bars are smaller than the symbols and therefore not visible

For the growth curve of CT-26 WT cells, 1 × 10E4 cells/cm^2^ were seeded in a CellScrew® mini. As previously shown, CT-26 WT cells adhered to the PLA surface within 24 h. Despite the extended attachment duration compared to B16-F10 cells, the cells had approximately doubled within the first 24 h (Fig. 7A). After 48 h, a 4.7-fold increase was observed, followed by an 8.5-fold increase after 72 h, and an 11.7-fold increase after 96 h. The harvest time after 96 h corresponds to a cell density of 11.7 ± 1.3 × 10E4 cells/cm^2^. During the cultivation, cell viability remains consistently above 92%. A complete medium change was carried out after 72 h, while glucose and lactate concentration in supernatant were measured daily (Fig. 7B). For CT-26 WT cells, Y_Lac/Glc_ reached 0.96 mol/mol over the cultivation period (0–72 h), indicating a mixed metabolic profile with a substantial glycolytic component.

To summarize growth kinetics across all three cell lines, populations doublings (PD) and specific growth rates (µ) were calculated for the respective exponential growth phases (Table 2). HEK-293 cells showed the longest doubling time (34.3 h), while B16-F10 and CT-26 WT cells doubled considerably faster (20.0 h and 23.3 h, respectively), in agreement with published values for these cell lines.

**Table 2:** Growth kinetics of HEK-293, B16-F10, and CT-26 WT cells cultivated in the CellScrew® minis

Cell line	System	Exponential phase [h]	PD	µ [h⁻^1^]	t_d [h]
HEK-293	CellScrew® mini	24–120	2.80	0.020	34.3
B16-F10	CellScrew® mini	24–96	3.59	0.035	20.0
CT-26 WT	CellScrew® mini	0–72	3.09	0.030	23.3

Population doublings (PD) and specific growth rates (µ) were calculated according to PD = log_2_ (N_t_/N_0_) and µ = ln(N_t_/N_0_)/(t − t_0_) over the respective exponential growth phases. Doubling time was determined as t_d_ = ln (2)/µ

### Reduction or replacement of detachment reagent

As the CellScrew® is made from bio-based PLA, the cultivation and harvesting of the cells should also focus on minimizing material usage to the greatest extent possible. Accutase is costly and often applied in excess, despite being favored for murine cells due to its gentle yet efficient detachment and preservation of cell viability and surface proteins (Lai et al. 2022). The goal was to determine the minimal amount of Accutase required for the detachment of B16-F10 and CT-26 WT cell lines.

In an initial experiment on polystyrene (PS) surfaces, Accutase was partially diluted in DPBS while maintaining a constant total volume. The plant-based dissociation reagent Envzyme™ Super was also evaluated for cell detachment. All conditions were harvested following the same protocol (Figure S1). For both cell lines, up to 80% of Accutase could be replaced with DPBS without significantly affecting harvest efficiency: B16-F10 cells were fully recovered, and 93% of CT-26 WT cells were harvested with only 20% Accutase, remaining within normal culture variability. B16-F10 cells were efficiently detached using Envzyme™ Super, reaching 100% harvest efficiency after 72 h. In contrast, CT-26 WT cells showed greater variability, achieving only 70% efficiency compared to 100% Accutase.

**Fig. 8:**
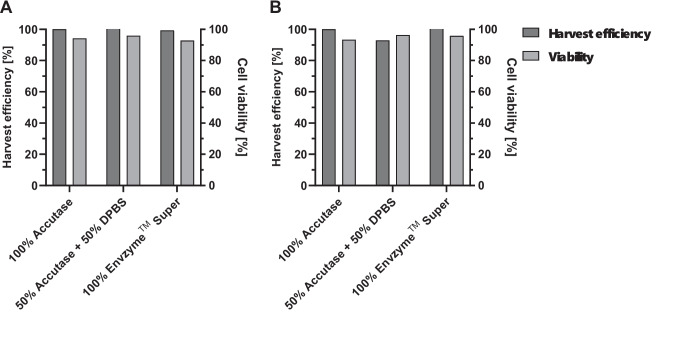
Reduction and replacement of dissociation reagent on PLA surface. Harvest efficiency [%] and cell viability [%] of B16-F10 (**A**) and CT-26 WT (**B**) cell lines after 96 h of culture on PLA surface in the CellScrew® mini. Cells were detached using 100% Accutase, 50% Accutase + 50% DPBS, or 100% Envzyme™ Super under identical conditions (*n* = 1)

In the next step, the reduction of Accutase and EnVzyme™ Super was tested on the PLA surface in the CellScrew® mini (Fig. 8). After 96 h of culture, B16-F10 (Fig. 8A) and CT-26 WT (Fig. 8B) cell lines were harvested using three protocols: 100% Accutase, 50% Accutase + 50% DPBS, and 100% EnVzyme™ Super. Despite the reduced Accutase concentration, 103% of B16-F10 cells and 93% of CT-26 WT cells were harvested. Using EnVzyme™ Super, 99% of B16-F10 cells and 107% of CT-26 WT cells recovered. Harvest efficiency values exceeding 100% can be attributed to ongoing cell proliferation during harvest procedure as well as to variability inherent to manual counting, and do not reflect an actual overestimation of recovered cells. Overall, these variations remain within the range of typical culture fluctuation. Thus, 50% Accutase or EnVzyme™ Super can be used as alternatives without affecting cell harvest efficiency. Moreover, cell viability remained above 93% for both cell lines post-harvest.

### Harvest efficiency of HEK-293

**Fig. 9:**
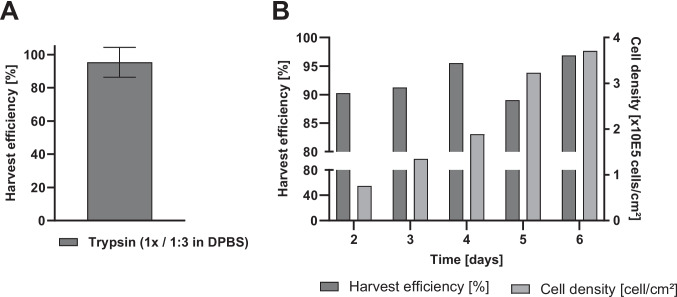
Reduction and harvest efficiency of dissociation reagent on PLA surface. (**A**) HEK-293 cells were seeded in CellScrew® mini and harvested using 100% trypsin or diluted trypsin (1:3 in DPBS), respectively. Harvest efficiency was normalized to 100% trypsin, *n* = 3 for each condition. (**B**) HEK-293 cells were seeded in CellScrew® 6 K at 6.6 × 10E4 cells/cm.^2^. At indicated time points, CellScrew® was harvested using diluted trypsin (1:3 in DPBS), and cell density was determined. Harvest efficiency was calculated by dividing the total cell number obtained after two harvests by the cell number from the first harvest, *n* = 1

HEK-293 cells are usually separated using trypsin, which is a common proteolytic enzyme used in cell culture. The reduction of trypsin concentration was tested using HEK-293 cells in the CellScrew® mini and CellScrew® 6 K. Figure 9A directly compares the harvest efficiency of diluted and undiluted Trypsin on HEK-293 in the CellScrew® mini. Dilution of trypsin with DPBS at a 1:3 ratio resulted in approximately 95% of the harvest efficiency of undiluted Trypsin, suggesting that, under the conditions tested, the amount of harvest enzyme may be reduced in the CellScrew® for HEK-293 cells. Whether comparable reductions can be achieved for other cell lines or at larger scales remains to be confirmed in dedicated follow-up studies.

In addition to significant cost and resource savings, cell morphology and characteristics are less affected when using lower trypsin concentrations (Brown et al. 2007). The harvest in Fig. 9A indicates similar efficiency (~ 95% ± 9%) when using reduced trypsin but does not take the cell density into account. Literature shows that high cell density and confluence influence the detachment rate, which subsequently leads to lower harvest efficiency when not considered (Deckers et al. 2022). Figure 9B shows the harvest efficiency of HEK-293 in the CellScrew® 6 K at increasing cell densities using diluted trypsin (1:3 in DPBS); these data were obtained from a single proof-of-concept run (n = 1) at the larger scale. While efficiency varied between 89 and 95% within this exploratory experiment, increasing cell density did not appear to substantially impair harvest performance under the conditions tested. A more comprehensive evaluation across additional cell lines and replicate runs would be required to generalize these observations.

## Discussion

The efficient scaling up of adherent cell cultures is hindered by the laborious handling required, the high susceptibility to process errors, and the limited surface area of conventional culture systems. Although microcarrier-based bioreactors offer greater surface areas, they can present challenges in terms of oxygen supply, shear sensitivity, and downstream processing. The CellScrew® platform addresses several of these limitations and has the potential to provide a scalable, robust, and resource-efficient solution for adherent mammalian cell culture.

Adequate oxygen supply is critical for high-density cell cultures. The k_L_a values measured across all three CellScrew® configurations were comparable to benchtop stirred-tank bioreactors and exceeded values reported for microcarrier-based systems (Sharma et al. 2022; Schwarz et al. 2020; Wyrobnik et al. 2025). As a k_L_a of 2.5 h⁻^1^ is considered sufficient for HEK-293 growth, the CellScrew® provides adequate oxygen transfer under the operating conditions and scales tested in this study, without apparent adverse effects on growth or viability (Boucher and Alves 1963).

In addition to oxygen supply, shear stress is a decisive factor influencing cell growth and viability in dynamic culture systems. The shear rates in the CellScrew® were comparable to microcarrier spinner cultures and far below levels reported to impair HEK-293 growth in TFF/ATF systems (Zhan et al. 2020). Within the rotational speeds and system sizes tested in this study, this low-shear environment, combined with decoupled mixing and oxygen transfer, supported stable attachment and viability of shear-sensitive adherent cells while enabling a compact and scalable culture format (Zhan et al. 2020; Zhang et al. 2023). Whether these favorable hydrodynamic conditions are maintained at further scaled-up configurations remains to be confirmed in dedicated follow-up studies.

The performance of the CellScrew® was further evaluated by comparing cell attachment and growth to conventional polystyrene (PS) tissue culture-treated roller bottle using HEK-293 cells. After 6 h, comparable attachment was observed across both systems. These values are in line with reports by Yang et al. who demonstrated attachment rates exceeding 95% for HEK-293 T and Vero cells after 4 h in Cytodex-1-based stirred cultures (Yang et al. 2019). Comparable attachment kinetics for HEK-293 and HEK-293 T cells have also been reported in fixed-bed bioreactor systems based on PET microfiber matrices, including the iCELLis Nano (Lesch et al. 2015; Stibbs et al. 2024a, b) and the Scale-X hydro platform (Leinonen et al. 2020), where adherent HEK-293(T) cells routinely establish stable cultures within the first hours after inoculation. The CellScrew® thus achieves attachment kinetics broadly in the same range as these fixed-bed systems, while operating on a flat PLA surface rather than a packed fibrous matrix. While microcarriers such as Cytodex 1 enable rapid attachment, they are associated with incomplete harvest of MSC even after 30 min of enzymatic incubation (Ng et al. 2021). In contrast, the CellScrew® offers high harvest efficiency using diluted Trypsin in DPBS (1:3), as shown in Fig. 9.

Beyond HEK-293, the murine CT-26 WT and B16-F10 cells also adhered to the PLA surface, albeit with distinct kinetics. The observed differences in attachment rates likely reflect intrinsic biological properties of the respective cell lines rather than system-related effects. Attachment kinetics of adherent cells depend on multiple factors, including cell type-specific integrin expression profiles, membrane composition, cell size, and the deposition of extracellular matrix proteins onto the cultivation surface (Khalili and Ahmad 2015). The highly metastatic B16-F10 melanoma cell line is known for its strong and rapid adhesion properties, consistent with its invasive phenotype (Fidler 1975), whereas CT-26 WT cells tend to display slower initial adhesion but robust subsequent proliferation. Importantly, all three cell lines reach near-complete attachment (> 98%) within 24 h, demonstrating that the CellScrew® system effectively supports the adhesion of cell lines with markedly different attachment profiles.

In addition, the complex three-dimensional architecture of the CellScrew® may influence both initial cell attachment and cell quantification in the supernatant, as cells are more readily retained within the structural features and on the PLA surface than on a smooth roller bottle surface. This could explain the apparently good initial attachment observed during the first hours, as well as the high standard deviation.

Metabolic analysis revealed patterns consistent with established models of cellular energy metabolism. An initial glycolytic overflow followed by partial lactate re-consumption or reduced lactate accumulation was observed, in agreement with reports by Liste-Calleja et al. *(*Liste-Calleja et al. 2015*).*

The combined evaluation of glucose and lactate levels provides deeper insight into cellular metabolic states: high glucose and high lactate levels are characteristic of the Warburg effect (Niepmann 2024), commonly observed in rapidly proliferating cells that favor glycolysis even under normoxic conditions. In contrast, high glucose with low lactate levels indicates a shift towards oxidative phosphorylation, typical of well-oxygenated cultures. Low glucose combined with high lactate, on the other hand, may reflect cellular stress or metabolite accumulation, potentially impairing cell function (Mulukutla et al. 2016).

Both mouse cancer cell lines investigated, CT-26 WT and B16-F10, exhibited a pronounced Warburg phenotype, characterized by high glucose consumption and increased lactate production, despite sufficient oxygen availability. The temporal decrease in glucose accompanied by increased lactate concentrations reflects their high glycolytic metabolism of the cells (Liberti and Locasale 2016). Importantly, the observed metabolite profiles followed expected physiological patterns and did not indicate abnormal accumulation, metabolic imbalance, or stress responses during cultivation in the CellScrew®. This supports the assumption that oxygen supply and mass transfer were sufficient across the tested scales. In mammalian cell cultures, lactate concentrations above approximately 20 mmol/L are generally considered growth-inhibitory (Lao and Toth 1997). Although cell line-specific thresholds have not been systematically reported for HEK-293, B16-F10, or CT-26 WT cells, the maximum lactate concentrations observed in the CellScrew® mini remained well below this threshold across all three cell lines, and glucose concentrations stayed above critical depletion levels during active growth. This indicates that the metabolic environment did not reach growth-limiting conditions and supports the interpretation that the observed growth kinetics and Y_Lac/Glc_ values reflect intrinsic cell line-specific metabolism rather than culture-induced stress.

Although both cell lines show slightly reduced growth on PLA compared to PS, they were successfully expanded with good cell yields in the CellScrew® minis. The cultivation of both cell lines showed comparable population doubling times (PDs) on PS and PLA surfaces, consistent with values reported in the literature—approximately 20 h for CT-26 WT cell line and 17 h for B16-F10 (Fidler 1975; Danciu et al. 2013). The CellScrew® mini integrates a total surface area of 850 cm^2^ within a compact flask format, effectively replacing nearly five standard 175 cm^2^ culture flasks. This consolidation substantially reduces handling effort, labor time, material consumption, and contamination risk.

Positioned in this landscape, the CellScrew® sits between conventional multilayer flasks and fixed-bed bioreactors. It offers continuous mixing, improved oxygen transfer, and a smaller footprint than multilayer vessels such as the Nunc™ Cell Factory™ or HYPERStack®, while requiring less capital and infrastructure than fixed-bed systems such as the iCELLis® Nano or Scale-X™ hydro platform (Lesch et al. 2015; Leinonen et al. 2020; Stibbs et al. 2024a), albeit at lower total surface area. Key limitations of the present study include the scales tested, the absence of viral vector productivity and long-term data, the slight growth reduction on PLA, the restricted cell-line panel (HEK-293, B16-F10, CT-26 WT), and the lack of a comprehensive life cycle assessment.

Taken together, no adverse effects on proliferation or viability were observed, and the characteristic fibroblast-like, spindle-shaped, and epithelial-like morphologies of CT-26 WT cells were preserved after re-growth on TC-treated flasks, indicating sustained cellular integrity. Overall, within the conditions evaluated in this study, the CellScrew® platform addresses several key parameters of conventional adherent cell culture by combining high oxygen transfer, low shear stress, efficient cell harvest, and scalable surface area. Its bio-based PLA construction further offers a reduced carbon footprint compared to petroleum-derived plastics such as polystyrene (Loughlin et al. 2024), while consolidating multiple vessels into a single compact device reduces material consumption and handling effort; a comprehensive life cycle assessment of the system represents an important direction for future work. From a forward-looking perspective, these results suggest that the CellScrew® could represent a promising step towards more standardized, resource-efficient, and scalable adherent manufacturing, with potential applications in cell-based therapies, vaccine production, and translational research, pending further validation in process-relevant settings.

From a future-perspective standpoint, further studies could explore the long-term performance of the CellScrew® platform with a wider variety of adherent cell types, including primary human cells and stem cells, as well as its potential integration into automated monitoring and control systems. Scaling the platform to even larger volumes and evaluating its compatibility with downstream processing workflows would also be needed further to assess its suitability for industrial and clinical applications. Such investigations would provide deeper insight into the versatility and robustness of the CellScrew® system and could support its broader implementation in high-demand bioprocessing environments.

## Supplementary Information

Below is the link to the electronic supplementary material.

Supplementary file1 (PDF 107 KB)

## Data Availability

All data supporting the findings of this study are available within the paper and its Supplementary Information.
