# Supplementary material for: Characterization and evaluation of a novel polylactic acid-made bioreactor for large-scale adherent cell expansion
Source: Appl Microbiol Biotechnol. 2026 May 22;110:155. doi: 10.1007/s00253-026-13880-4 (PMC13197453; doi:10.1007/s00253-026-13880-4)
Supplement: Supplementary file 1 [file 253_2026_13880_MOESM1_ESM.pdf]

Title: **Characterization and evaluation of a novel polylactic acid-made bioreactor for large-scale adherent cell expansion**

Applied Microbiology and Biotechnology

Submission to Special Collection: Single Use technologies in Bioprocessing

Author information:

Björn Boshof[1, §], Alena Hüppner[2,§], Johanna Eichberg[1], Henning Reyer[1], Lukas Käßer[1, *], Anne Kölsch[2, *]

1 GreenElephant Biotech GmbH, Giessen, Germany

2 TRON – Translational Oncology at the University Medical Center of the Johannes Gutenberg University gGmbH, Mainz, Germany

§ These authors contributed equally

* These authors contributed equally as senior authors

Email address of corresponding author:

bjoern@greenebt.com , Anne.Koelsch@trOn-Mainz.de

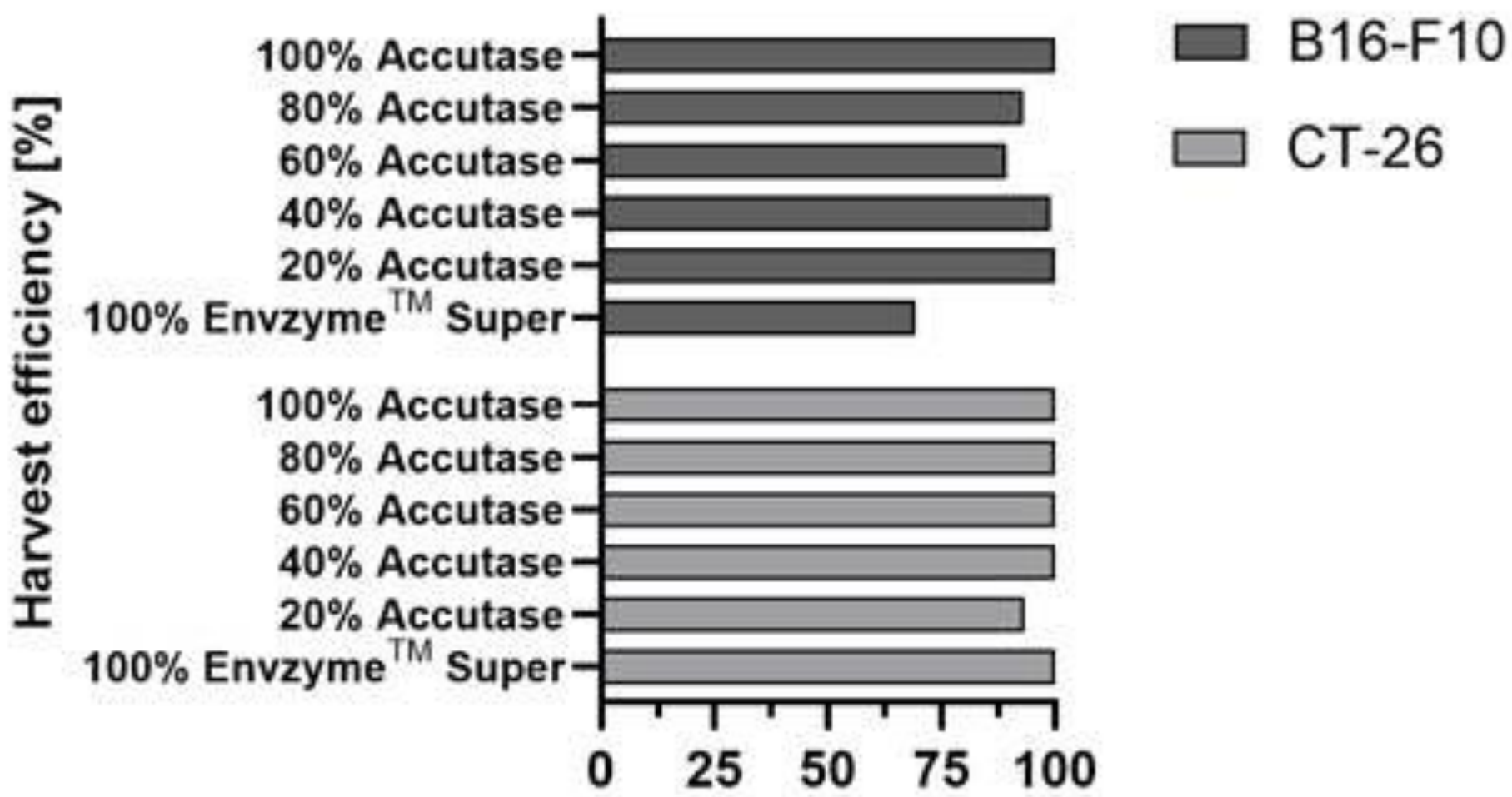

Figure S1. Reduction and replacement of dissociation reagent on PLA surface. Harvest efficiency [%] normalized to 100% Accutase after 72 hours of cultivation on PS surface and subsequent detachment with Accutase (100–20%) or Envzyme™ Super (n=2).
